# Potentiation of 17β-estradiol synthesis in the brain and elongation of seizure latency through dietary supplementation with docosahexaenoic acid

**DOI:** 10.1038/s41598-017-06630-0

**Published:** 2017-07-24

**Authors:** Yasuhiro Ishihara, Kouichi Itoh, Miki Tanaka, Mayumi Tsuji, Toshihiro Kawamoto, Suguru Kawato, Christoph F. A. Vogel, Takeshi Yamazaki

**Affiliations:** 1 Laboratory of Molecular Brain Science, Graduate School of Integrated Arts and Sciences, Hiroshima University, Hiroshima, 739-8521 Japan; 2 Center for Health and the Environment, University of California, Davis, Davis California, 95616 USA; 3 Laboratory for Brain Science, Kagawa School of Pharmaceutical Sciences, Tokushima Bunri University, Kagawa, 769-2193 Japan; 4 Department of Environmental Health, University of Occupational and Environmental Health, Fukuoka, 807-8555 Japan; 5 Department of Biophysics and Life Sciences, Graduate School of Arts and Sciences, University of Tokyo, Tokyo, 153-8902 Japan; 6 Department of Urology, Juntendo University, Graduate School of Medicine, Tokyo, 113-8431 Japan; 7 Department of Environmental Toxicology, University of California, Davis, Davis California, 95616 USA

## Abstract

Several studies have shown that docosahexaenoic acid (DHA) attenuates epileptic seizures; however, the molecular mechanism by which it achieves this effect is still largely unknown. DHA stimulates the retinoid X receptor, which reportedly regulates the expression of cytochrome P450 aromatase (P450arom). This study aimed to clarify how DHA suppresses seizures, focusing on the regulation of 17β-estradiol synthesis in the brain. Dietary supplementation with DHA increased not only the expression of P450arom, but also 17β-estradiol in the cerebral cortex. While DHA did not affect the duration or scores of the seizures induced by pentylenetetrazole, DHA significantly prolonged the seizure latency. A P450arom inhibitor, letrozole, reduced 17β-estradiol levels and completely suppressed the elongation of seizure latency elicited by DHA. These results suggest that DHA delays the onset of seizures by promoting the synthesis of 17β-estradiol in the brain. DHA upregulated the expression of anti-oxidative enzymes in the cerebral cortex. The oxidation in the cerebral cortex induced by pentylenetetrazole was significantly attenuated by DHA, and letrozole completely inhibited this suppressive action. Thus, the anti-oxidative effects of 17β-estradiol may be involved in the prevention of seizures mediated by DHA. This study revealed that 17β-estradiol in the brain mediated the physiological actions of DHA.

## Introduction

The n-3 polyunsaturated fatty acid docosahexaenoic acid (DHA) is a major fatty acid in the brain. Recently, a DHA transporter—major facilitator superfamily domain-containing protein 2A, which actively delivers esterified DHA to the brain—was identified^1^. However, Chen *et al*. reported that the DHA in the brain is derived from the unesterified plasma DHA pool^2^. Thus, it is thought that while the brain incorporates esterified DHA via major facilitator superfamily domain-containing protein 2A, unesterified DHA crosses through the blood-brain barrier.

The brain has long been considered a target organ of steroid hormones that are secreted from the peripheral endocrine system, but a recent study shows that the brain synthesizes and secretes steroid hormones (neurosteroids) to maintain a range of neuronal functions. Neurosteroids are reported to induce dendrite development^3^, and we have shown that neurosteroids are involved in synaptogenesis^4^ and spinogenesis^5^. In addition, we found that the representative neurosteroids 17β-estradiol and progesterone suppress neuronal injury induced by reperfusion following ischemia as well as by environmental chemicals, such as tributyltin and methyl mercury^6–9^.

The neurosteroid 17β-estradiol is most effective in terms of neuroprotection, and it exerts protective effects against a range of neurological disorders—from neurodegenerative to psychiatric diseases^10, 11^. 17β-estradiol is synthesized from testosterone metabolized by cytochrome P450 aromatase (P450arom) (Supplementary Fig. S1). We previously reported that 9-cis retinoic acid, an endogenous agonist of the retinoid X receptor (RXR), stimulated the upregulation of P450arom and, subsequently, increased the levels of 17β-estradiol in rat hippocampal slice cultures^12^. This result suggests that RXR plays a fundamental role in the regulation of 17β-estradiol synthesis in the brain. Interestingly, DHA is known to act as an RXR agonist^13^ and may, therefore, activate the synthesis of 17β-estradiol in the brain. Taken together, these findings suggest that 17β-estradiol may mediate the actions of DHA in the brain.

Estrogens have long been considered to exacerbate epileptic seizures^14^. This is in contrast to progesterone, which is well known to have anticonvulsant effects^15, 16^ caused by the binding of progesterone metabolite allopregnanolone to γ-aminobutyric acid A (GABA_A_) receptors and eliciting an inhibitory current^17^. However, it was recently observed that the effect of estrogens on epilepsy may be concentration dependent, since higher concentrations of estrogens were found to exacerbate seizures, while lower concentrations had an anti-convulsant effect^18^. Furthermore, estrogens have been reported to decrease the mortality rate from epileptic seizures^19^. Thus, endogenous 17β-estradiol may play a role in protecting neurons from the excitotoxicity that accompanies seizures.

A clinical trial showed that DHA intake decreased the frequency of seizures in epileptic subjects^20^, and that the anti-convulsant effects of DHA may occur in a dose-dependent manner^21^. As described above, DHA is considered to increase brain levels of 17β-estradiol, which can suppress epileptic seizures via RXR activation. Therefore, in the present study, we investigated the effects of DHA on seizures, focusing on the dynamics of 17β-estradiol in the brain.

## Results

### Dietary supplementation with DHA increases the expression of P450arom and 17β-estradiol

The fatty acid content of soybean and cottonseed oils was fairly similar but the amount of linolenic acid in cottonseed oil was 1/16 (0.4% [w/w]) of the amount in soybean oil (6.7% (w/w): Table 1). Cottonseed oil contain much less n-3 polyunsaturated fatty acid than linolenic acid. Cottonseed oil contained a lot of linoleic acid, but mammals do not convert linoleic acid to linolenic acid. Thus, a diet in which cottonseed oil was used as a lipid was employed as an n-3 polyunsaturated fatty acid-deficient diet.

**Table 1:** Fatty acid content of soybean and cottonseed oils.

		Soybean oil	Cottonseed oil
Decanoic acid	C10:0	0	0
Lauric acid	C12:0	0	10
Myristic acid	C14:0	63	560
Myristoyleic acid	C14:1	0	0
Pentadecanoic acid	C15:0	11	13
Pentadecenoicacid	C15:1	0	0
Palmitic acid	C16:0	10000	18000
Palmitoleic acid	C16:1	87	600
Heptadecanoic acid	C17:0	85	71
Heptadecenoic acid	C17:1	0	0
Stearic acid	C18:0	3800	2300
Oleic acid	C18:1	22000	17000
Linoleic acid	C18:2 n-6	52000	56000
Linolenic acid	C18:3 n-3	6700	420
Gamma-linolenic acid	C18:3 n-6	30	0
Arachidic acid	C20:0	310	230
Eicosenoic acid	C20:1	180	75
Eicosadienoic acid	C20:2 n-6	32	9
Eicosatrienoic acid	C20:3 n-6	8	11
Arachidonic acid	C20:4 n-6	0	0
Eicosapentaenoic acid	C20:5 n-3	0	0
Behenic acid	C22:0	330	110
Docosenic acid	C22:1	0	10
Docosadienoic acid	C22:2	0	0
Docosatetraenoic acid	C22:4	0	0
Docosapentaenoic acid	C22:5 n-3	0	0
Docosahexaenoic acid	C22:6 n-3	0	110
Lignoceric acid	C24:0±	130	91

(*mg/100* *g oil*).

The detection limit was 1 mg/100 g oil.

In the present study, mice were fed a diet containing soybean oil, cottonseed oil or cottonseed oil supplemented with DHA for 28 days. Body weight and food intake among the three diet groups did not differ over the course of the study (Supplementary Fig. S2). Furthermore, no marked weight differences for the whole brain, cerebral cortex, cerebellum or hippocampus were found among the three groups after 28 days of feeding (Supplementary Fig. S2).

**Figure 1:**
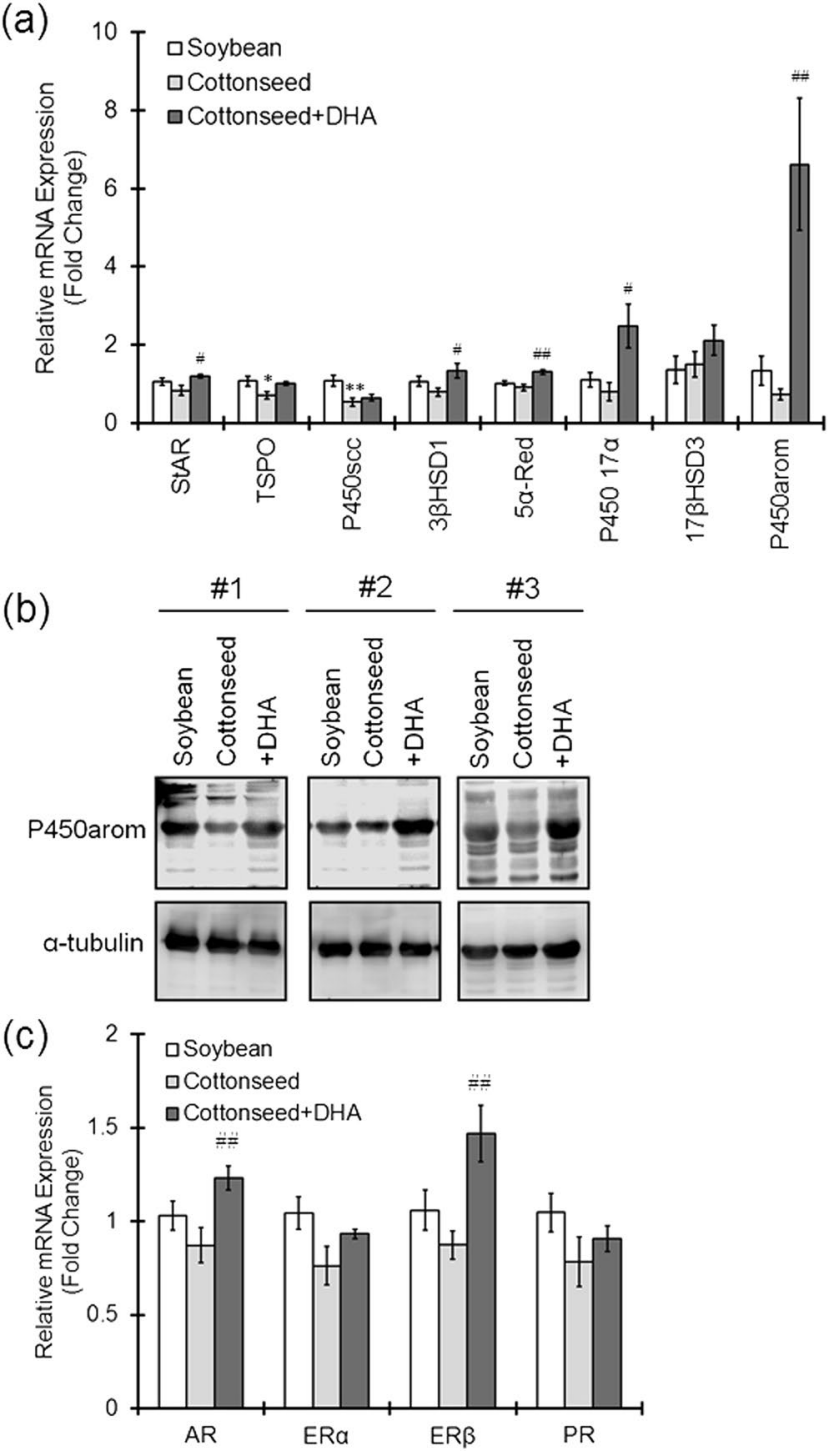
Upregulation of P450arom in the brain by DHA dietary supplementation. Mice were fed a diet that contained soybean oil, cottonseed oil or cottonseed oil supplemented with DHA for 28 days. (**a**) The mRNA expression of steroid-synthesizing enzymes in the cerebral cortex was measured by real-time PCR. The levels of mRNA are represented as the fold-change in comparison to the soybean oil group. The values represent the mean ± S.E. (n = 9 animals in each group). Data were analyzed using a one-way ANOVA, followed by Tukey’s test. ^*^P < 0.05 and ^**^P < 0.01 vs. soybean oil group, ^#^P < 0.05 and ^##^P < 0.01 vs. cottonseed oil group. StAR, steroidogenic acute regulatory protein; TSPO, translocator protein; P450scc, cytochrome P450 cholesterol side chain cleavage enzyme; 3βHSD1, 3β-hydroxysteroid dehydrogenase type 1; 5α-Red, 5α-reductase; P450 17α, cytochrome P450 17α; 17βHSD3, 17 β-hydroxysteroid dehydrogenase type 3, P450arom, cytochrome P450 aromatase. (**b**) P450arom expression was evaluated by immunoblotting. The results of three independent experiments (#1 to #3) are shown. (**c**) The mRNA expression of steroid hormone receptors in the cerebral cortex was measured by real-time PCR. The values represent the mean ± S.E. (n = 9 animals in each group). Data were analyzed using a one-way ANOVA, followed by Tukey’s test. ^##^P < 0.01 vs. cottonseed oil group. AR, androgen receptor, ERα, estrogen receptor α, ERβ, estrogen receptor β; PR, progesterone receptor.

It has been reported that DHA can act as an agonist of RXR^13^ and rat hippocampus slices exposed to RXR in culture have increased 17β-estradiol synthesis^12^. Thus, mRNA expression of steroid-synthesizing enzymes was measured in the cerebral cortex of mice fed each of the three diets. Levels of translocator protein and cytochrome P450 cholesterol side chain cleavage enzyme mRNA in the cottonseed oil diet group were significantly lower in comparison to the soybean oil diet group (Fig. 1a). Mice fed the cottonseed oil diet tended to have lower P450arom mRNA levels than mice fed the soybean oil diet (Fig. 1a), and this result was mirrored by Western blot analysis (Fig. 1b). DHA supplementation significantly increased the mRNA expression of steroidogenic acute regulatory protein, 3β-hydroxysteroid dehydrogenase type 1, 5α-reductase, cytochrome P450 17α and P450arom (Fig. 1a). DHA supplementation largely enhanced the P450arom protein expression (Fig. 1b). The levels of androgen receptor and estrogen receptor β mRNA were slightly (but significantly) elevated by DHA supplementation (Fig. 1c).

**Figure 2:**
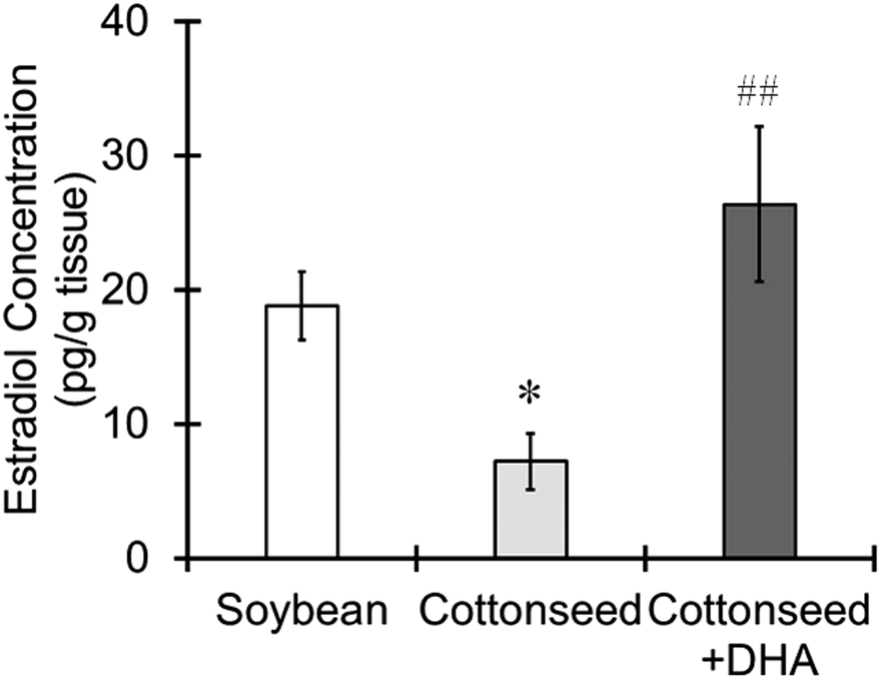
Increased 17β-estradiol levels in the brain by DHA dietary supplementation. Mice were fed a diet containing soybean oil, cottonseed oil or cottonseed oil supplemented with DHA for 28 days. The cerebral cortex was then removed, and the 17β-estradiol levels were determined by EIA. The values represent the mean ± S.E. (n = 7 animals in each group). Data were analyzed using a one-way ANOVA, followed by Tukey’s test. ^*^P < 0.05 vs. soybean oil group, ^##^P < 0.01 vs. cottonseed oil group.

Because P450arom catalyzes the reaction in which 17β-estradiol is synthesized from testosterone (Supplementary Fig. S1), the concentration of 17β-estradiol was determined in the cerebral cortex. Mice fed the cottonseed oil diet had a significantly lower concentration of 17β-estradiol than mice fed the soybean oil diet (Fig. 2). Of note, DHA supplementation to the cottonseed oil diet resulted in a significantly increased concentration of 17β-estradiol (Fig. 2). These results suggest that DHA dietary supplementation enhances the synthesis of 17β-estradiol in the brain via the upregulation of the P450arom expression.

### The suppressive effects of DHA on seizures

Epilepsy is a chronic neurological disorder that is accompanied by convulsive seizures, which are elicited by abnormal neuronal activity. There is evidence that neurosteroid balance is an important factor in the onset and/or progression of epilepsy; progesterone is well known to suppress convulsive seizures^15, 16^, and recently, there is increasing evidence to suggest that 17β-estradiol can attenuate convulsive seizures^18^. Thus, the effect of DHA on acute seizures induced by single dose of the chemical convulsant pentylenetetrazole (PTZ) was examined next.

**Figure 3:**
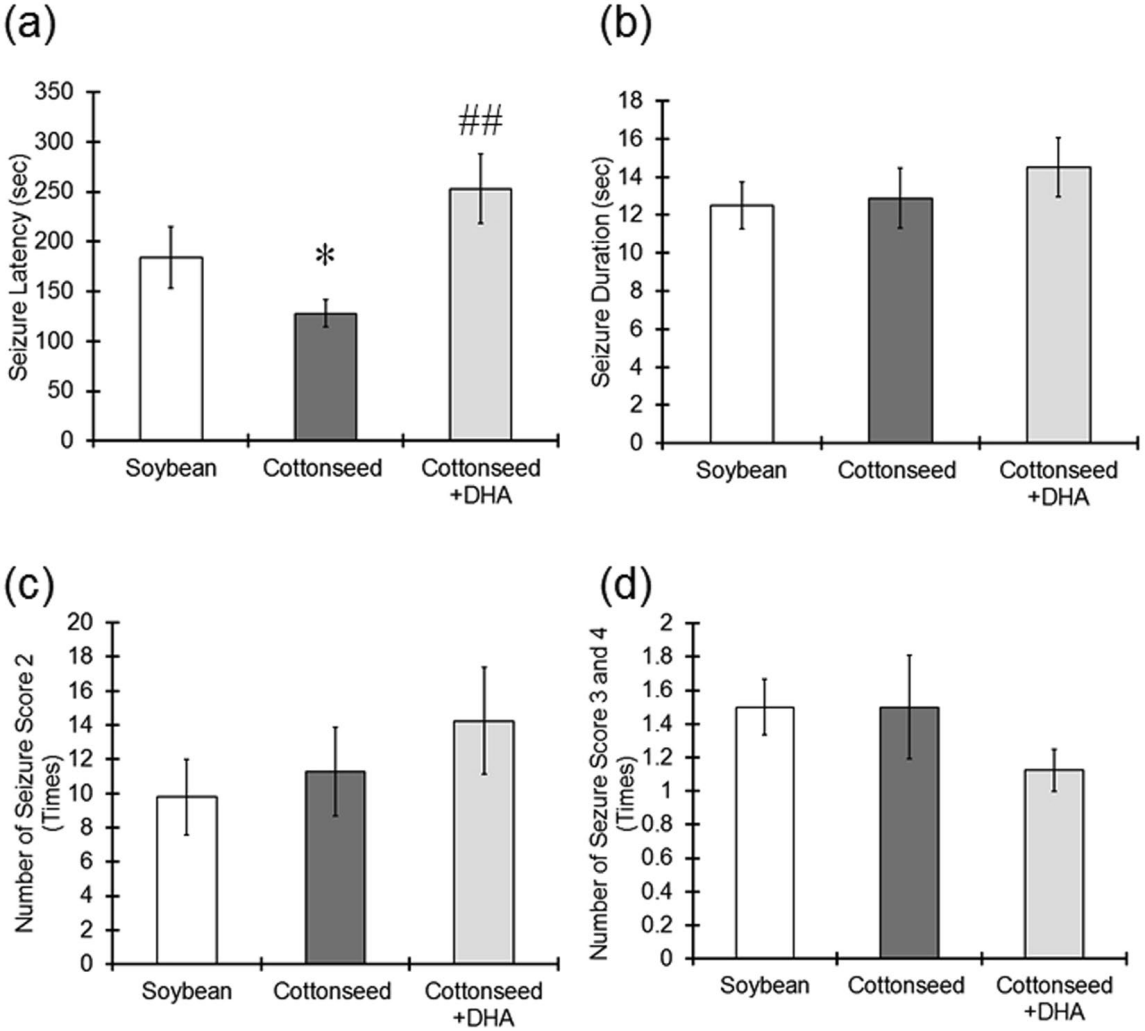
Elongation of the seizure latency by DHA dietary supplementation. Mice were fed a diet containing soybean oil, cottonseed oil or cottonseed oil supplemented with DHA for 28 days. PTZ (60 mg/kg) was intraperitoneally injected, and the convulsive behaviors of the mice were observed for 15 minutes to evaluate (**a**) the seizure latency, (**b**) the seizure duration and (**c**,**d**) the seizure score. The values represent the mean ± S.E. (n = 7–9 animals in each group). Data were analyzed using a one-way ANOVA, followed by Tukey’s test. ^*^P < 0.05 vs. soybean oil group, ^##^P < 0.01 vs. cottonseed oil group.

All mice treated with PTZ had seizures. While there were no marked differences in seizure duration or scores between mice fed a soybean oil diet and those fed a cottonseed oil diet, seizure latency in the cottonseed oil group was shorter than that in the soybean oil group (Fig. 3a–d). Dietary supplementation with DHA significantly prolonged the seizure latency in comparison to mice fed the cottonseed oil diet alone (Fig. 3a). However, DHA supplementation did not affect the seizure duration or score (Fig. 3b–d).

Letrozole, a P450arom inhibitor, was used to determine whether brain 17β-estradiol is involved in the elongation of seizure latency induced by DHA supplementation. A preliminary study of concentration over time was done with an intraperitoneal injection of letrozole (10 mg/kg). Three days after administration, letrozole was nearly undetectable in the cerebral cortex, cerebellum, hippocampus and serum (Supplementary Fig. S3). Thus, it was determined that the concentration of letrozole in the brain would remain above the IC_50_ needed for P450arom (11.5 nM)^22^ provided that it was administered every other day. This dosing schedule did not noticeably affect body weight, food intake or brain weight of the mice.

**Figure 4:**
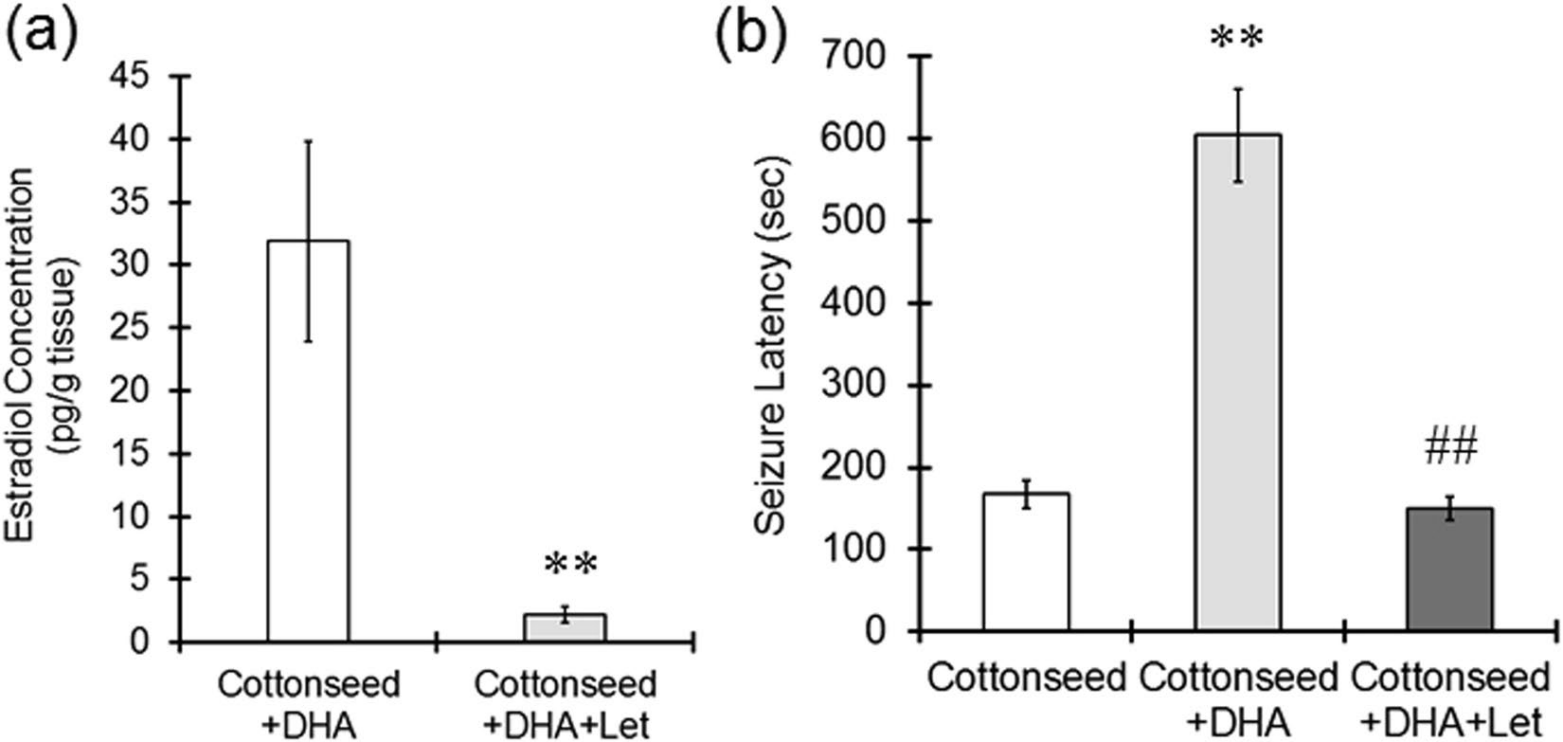
Letrozole reduced 17β-estradiol levels in the brain and shortened seizure latency. Mice were fed a diet containing cottonseed oil or cottonseed oil supplemented with DHA and injected with letrozole (10 mg/kg) intraperitoneally every other day for 28 days. (**a**) The 17β-estradiol levels in the cerebral cortex were determined by EIA. The values represent the mean ± S.E. (n = 7 animals in each group). Data were analyzed using Student’s *t*-test. ^**^P < 0.01 vs. cottonseed oil plus DHA group. (**b**) PTZ (60 mg/kg) was intraperitoneally injected, and the convulsive behaviors of the mice were observed for 15 minutes to evaluate the seizure latency. The values represent the mean ± S.E. (n = 8–10 animals in each group). Data were analyzed using a one-way ANOVA, followed by Tukey’s test. ^**^P < 0.01 vs. cottonseed oil group, ^##^P < 0.01 vs. cottonseed oil plus DHA group.

Letrozole treatment markedly decreased 17β-estradiol levels in the cerebral cortex of animals that received the cottonseed oil diet supplemented with DHA (Fig. 4a). Because the 17β-estradiol value measured was around the detection limit of the enzyme-linked immunosorbent assay (EIA), we confirmed the 17β-estradiol level using liquid chromatography-tandem mass spectrometry (LC/MS/MS). The 17β-estradiol level in the cerebral cortex of the letrozole-treated mice was 0.52 pg/g tissue based on the LC/MS/MS analysis, which confirmed that letrozole treatment resulted in a large reduction in the 17β-estradiol level. Letrozole treatment also significantly decreased seizure latency elicited by DHA supplementation (Fig. 4b) but did not affect seizure duration or score. Thus, increasing 17β-estradiol levels via dietary supplementation with DHA may prolong the latency of seizures induced by PTZ.

The results shown in Figs 3 and 4b are from two independent experiments. Both experiments indicate that seizure latency increased by approximately 2.5 times when animals consumed cottonseed oil supplemented with DHA. While the reason for this result is unclear, the data strengthens the conclusion that DHA supplementation can prolong seizure latency.

### The anti-oxidative effects of 17β-estradiol synthesis by DHA

We previously reported that 17β-estradiol protects hippocampal neurons by suppressing oxidative stress^8^, and stimulation of microglial estrogen receptors attenuates excessive activation of microglia^23^. Because oxidative stress and/or neuroinflammation elicited by activated microglia can modulate seizures^24, 25^, the effect of DHA on brain oxidative stress and microglial activity was evaluated. The following anti-oxidative and detoxification enzyme mRNA levels were measured in the cerebral cortex: superoxide dismutase 1, superoxide dismutase 2, glutathione peroxidase 1, catalase, glutathione reductase, glutathione S-transferase (GST) A3, GSTM1 and M-GST.

**Figure 5:**
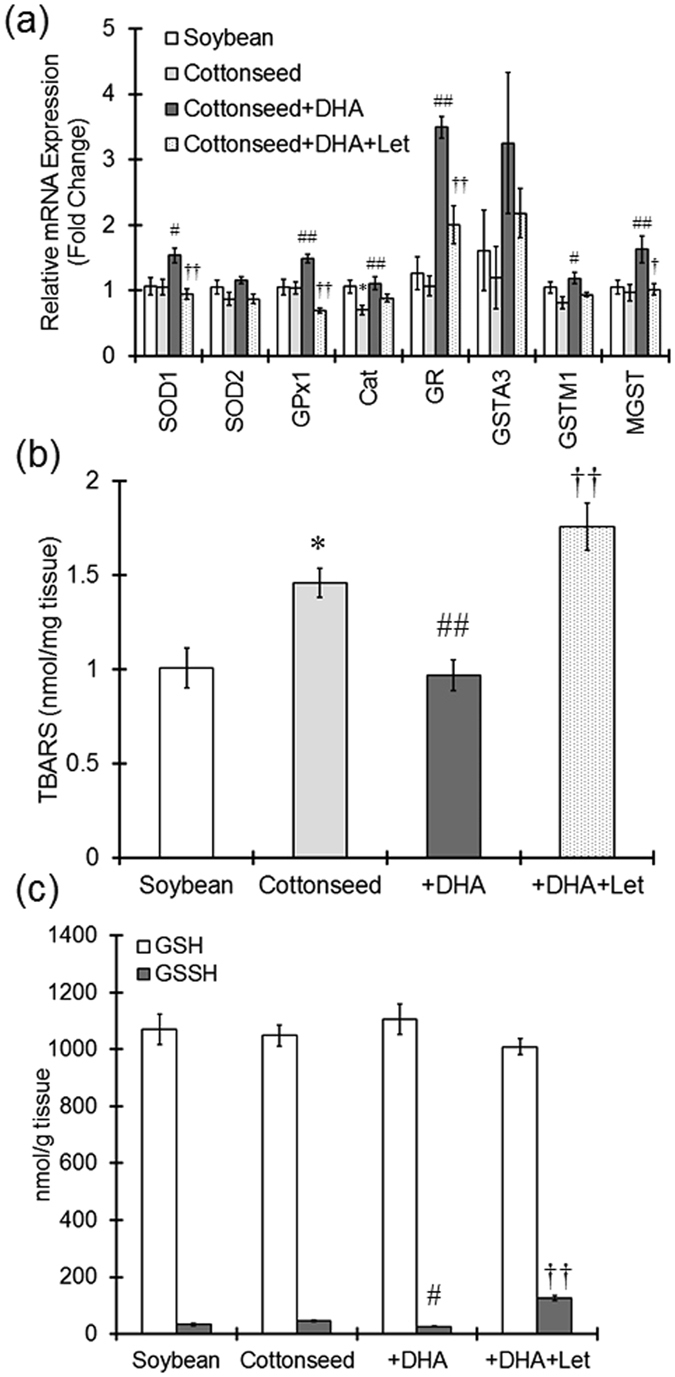
Suppression of PTZ-elicited oxidative stress by DHA dietary supplementation. Mice were fed a diet containing soybean oil, cottonseed oil, cottonseed oil plus DHA for 28 days. Letrozole (10 mg/kg) was intraperitoneally injected every other day. (**a**) The mRNA expression of anti-oxidative and metabolizing enzymes in the cerebral cortex was measured by real-time PCR. Levels of mRNA are represented as the fold-change in comparison to the soybean oil group. The values represent the mean ± S.E. (n = 9–10 animals in each group). Data were analyzed using a one-way ANOVA, followed by Tukey’s test. ^*^P < 0.05 vs. soybean oil group, ^#^P < 0.05 and ^##^P < 0.01 vs. cottonseed oil group, ^†^P < 0.05 and ^††^P < 0.01 vs. cottonseed oil plus DHA group. SOD1, superoxide dismutase 1; SOD2, superoxide dismutase 2; GPx1, glutathione peroxidase 1; Cat, catalase; GR, glutathione reductase; GSTA3, glutathione S-transferase A3; GSTM1, glutathione S-transferase M1; MGST, microsomal glutathione S-transferase. The amount of lipid peroxide (**b**) and the GSH and GSSG levels (**c**) in the cerebral cortex were determined at 3 h after the administration of PTZ. The values represent the mean ± S.E. (n = 7–10 animals in each group). Data were analyzed using a one-way ANOVA, followed by Tukey’s test. ^*^P < 0.05 vs. soybean oil group, ^#^P < 0.05 and ^##^P < 0.01 vs. cottonseed oil group, ^†^P < 0.05 and ^††^P < 0.01 vs. cottonseed oil plus DHA group.

Catalase mRNA expression from the cottonseed oil diet alone was significantly reduced compared to the soybean oil diet (Fig. 5a). The mRNA expression of superoxide dismutase 1, glutathione peroxidase 1, catalase, glutathione reductase, GSTM1 and MGST was significantly upregulated by DHA supplementation. However, the increased expression of superoxide dismutase 1, glutathione peroxidase 1, glutathione reductase, and MGST was significantly suppressed by treatment with letrozole. These results suggest that DHA-induced 17β-estradiol levels are involved in upregulating the expression of anti-oxidative and detoxification enzymes.

Microglial phagocytic activity was examined by Iba1/CD68 double-staining^26^. No marked differences were observed in microglial morphology or CD68 expression among the three diet groups (Supplementary Fig. S4). In addition, mRNA expression of the inflammatory molecules interleukin-1β and inducible nitric oxide synthase in the cerebral cortex showed no marked differences among the three diet groups (Supplementary Fig. S5). The mRNA expression of glial fibrillary acidic protein and S100β (markers of astrocyte activity) were also similar among the three diet groups (Supplementary Fig. S5). Thus, it was concluded that DHA did not affect the levels of microglial or astrocytic activity under our experimental conditions.

Oxidative stress is known to be involved in seizures elicited by PTZ^27^. Because DHA upregulated the expression of anti-oxidative enzymes, lipid peroxides in the cerebral cortex was measured as a reflection of oxidative stress induced by PTZ. Animals that received the cottonseed oil diet had significantly higher levels of lipid peroxide than those that received the soybean oil diet (Fig. 5b). Dietary supplementation with DHA clearly decreased the levels of lipid peroxide, while co-treatment with DHA and letrozole largely potentiated lipid peroxidation (Fig. 5b). Supplementation of the cottonseed oil diet with DHA significantly decreased cerebral cortex oxidized glutathione (GSSG) levels compared to cottonseed oil only diet (Fig. 5c). Letrozole treatment significantly increased GSSG levels compared to no letrozole treatment. These results indicate that DHA supplementation increases 17β-estradiol levels and that this may attenuate the oxidative stress induced by PTZ.

## Discussion

When mice were fed an n-3 polyunsaturated fatty acid-deficient diet supplemented with DHA, P450arom expression and 17β-estradiol levels in the cerebral cortex were highly elevated. Because P450arom is an enzyme that synthesizes 17β-estradiol from testosterone, the increases in 17β-estradiol are most likely due to the upregulation of P450arom. It has been reported that DHA can act as an agonist of RXR^13^. Our promoter analysis showed that the P450arom promoter has several half sites of the RXR binding region. Thus, RXR activated by DHA might directly elicit P450arom transactivation.

The latency of seizures induced by PTZ was significantly prolonged by the intake of DHA, and this elongation was clearly suppressed by the administration of the P450arom inhibitor letrozole. DHA may induce 17β-estradiol synthesis in the brain and, therefore, contribute to the elongation of seizure latency.

DHA enhances the expression of anti-oxidative enzymes and GST. Organic hydroperoxides and 4-hydroxyalkenals, which are found inside cells, act as substrates for GST. Organic hydroperoxides are well-known to induce oxidative stress followed by lipid peroxidation, and 4-hydroxyalkenals are reported to produce reactive oxygen species, mainly superoxide anions and hydrogen peroxides, leading to oxidative injury^28^. The onset of seizure is considered to be closely related to oxidative stress^24^. Thus, the anti-oxidative effects of 17β-estradiol may be part of the elongation of seizure latency mediated by DHA.

DHA has several physiological actions, including anti-oxidative activity, but its molecular mechanisms are largely unknown. In the present study, we showed that DHA increased the level of 17β-estradiol in the brain via the upregulation of P450arom. 17β-estradiol has been suggested to have neuroprotective effects because the expression of P450arom is elevated after ischemia^29^ and because P450arom KO mice show more severe neuronal injury after ischemia in comparison to wild-type mice^30^. We have shown that 17β-estradiol protects hippocampal neurons from ischemia and environmental chemicals^6, 8^. 17β-estradiol might be protective by suppressing oxidative stress. Both genomic and non-genomic pathways are thought to mediate the anti-oxidative action of 17β-estradiol^31^. For example, 17β-estradiol has been reported to attenuate oxidative stress via upregulation of anti-oxidative enzymes superoxide dismutase 1^32^, superoxide dismutase 2^33^, glutathione peroxidase 1^34^ and catalase^34^. Furthermore, 17β-estradiol can suppress nuclear factor-κB activity contributing to the transcription of a number of pro-inflammatory molecules^35^. In the case of non-genomic anti-oxidation pathways, 17β-estradiol is related to activating kinase signaling, regulating the rate of mitochondrial uncoupling, directly eliminating reactive oxygen species, and attenuating the inflammatory reaction of the microglia^7, 23, 36^. Because DHA upregulated the expression of anti-oxidative enzymes, which was attenuated by treatment with letrozole in the present study, synthesized 17β-estradiol is considered to be involved in the anti-oxidative effects of DHA. Although the involvement of non-genomic pathways of 17β-estradiol in DHA-induced neuroprotection is unclear, genomic signaling, upregulation of anti-oxidative enzymes, induced by 17β-estradiol could be one of neuroprotective mechanisms of DHA.

DHA administration just before PTZ treatment is reported to suppress seizure onset^37, 38^. The effects of DHA on the neuronal Na^+^ and Ca^2+^ channels have been proposed as acute actions of DHA. Vreugdenhil *et al*. demonstrated that treatment of rat primary hippocampal neurons with DHA produced a concentration-dependent shift in the voltage dependence of the inactivation of both Na^+^ and Ca^2+^ current to more hyperpolarized potentials^39^, indicating that the inactivation was accelerated and that the recovery from inactivation was slowed. This effect of DHA might reduce neuronal excitability and have an anti-convulsive effect. In addition, DHA has been shown to enhance the uptake of aspartate via glutamate transporter-1 and excitatory amino acid carrier 1^40^. GABA_A_ receptor function can also be modulated by DHA; a low concentration of DHA can accelerate the desensitization after the peak GABA-induced current is reached, while a high DHA concentration can suppress the peak amplitude of the GABA response^41^. These effects of DHA on channels and/or transporters might be involved in the acute suppressive action against seizures. In the present study, we showed that the DHA intake contributed to the elongation of the seizure latency. Chronic treatment with letrozole almost completely suppressed 17β-estradiol synthesis as well as the elongation of latency induced by DHA intake, indicating that 17β-estradiol synthesized via DHA could be closely involved in the elongation of seizure latency. This action is a chronic effect of DHA because the induction of P450arom contributes to increases in the 17β-estradiol levels in the brain. Thus, the mechanism that we demonstrated in the present study may represent one of the mechanisms of action related to the chronic effects of DHA.

Clinical and animal studies show that DHA intake can prevent convulsive seizures^21^. However, the protective effect of DHA may be dependent on the amount and duration of DHA intake as well as seizure models used^20, 42, 43^. Willis *et al*. reported that DHA has no anti-convulsant effects against PTZ-induced seizures in ICR mice^44^; they examined the threshold for myoclonic seizures and the tonic extension using an intravenous PTZ infusion test in ICR mice that were fed DHA for 4 weeks and concluded that DHA did not affect the threshold for PTZ seizures. On the other hand, we observed that the onset latency and duration of the first generalized clonic convulsions were affected by a single dose of PTZ (60 mg/kg). After single-dose injections of PTZ, two types of seizures, non-convulsive (scores 1 and 2) and convulsive motor seizures (scores 3 and 4), occurred, but tonic convulsions never occurred with this dose of PTZ. There are several possible explanations for the discrepancy between the present study and the one done by Willis *et al*. other than the above-described differences in the conditions of the seizure experiments. They include: (1) Purity of DHA. We used DHA with >90% purity, while the purity of the DHA used by Willis *et al*. was 70%. (2) Diet preparation. Willis *et al*. dried their DHA-containing chow at 80 °C for 2 h. Unsaturated fatty acids, including DHA, are sensitive to oxidation, and heat treatment potentiates their oxidation. We rapidly mixed AIN-93G with DHA and then maintained the chow at −30 °C until use.

In the present study, 17β-estradiol actively synthesized by DHA prolonged seizure latency, and the attenuation of oxidative stress was suggested to be one of the protective mechanisms of 17β-estradiol. Olivetti *et al*. reported that 17β-estradiol prevented spasms in infancy by restoring depleted interneuron populations using a mouse model of X-linked infantile spasms syndrome^45^. However, Kurt *et al*. showed that estrogen signals mediated by G protein-coupled estrogen receptor 1 increased the development of PTZ kindling^46^, indicating that the effects of 17β-estradiol on seizures might depend on a downstream pathway. Further studies are needed to clarify the protective mechanism of DHA and 17β-estradiol against convulsive seizures.

In conclusion, we found that dietary supplementation with DHA upregulates P450arom expression, subsequently increasing the level of 17β-estradiol in the cerebral cortex. DHA-induced 17β-estradiol synthesis can suppress convulsive seizures via its anti-oxidative effects. The present study suggests that 17β-estradiol in the brain mediates the physiological actions of DHA.

## Methods

### Materials

Docosahexaenoic acid (DHA; >90% purity) was purchased from Larodan (Solna, Sweden). Letrozole was obtained from Tokyo Chemical Industry (Tokyo, Japan). Pentylenetetrazole (PTZ) was purchased from Sigma-Aldrich (St. Louis, MO, USA). All other chemicals were obtained from Wako Pure Chemical Industries (Osaka, Japan), Nacalai Tesque (Kyoto, Japan) or Sigma-Aldrich and were of reagent grade.

### Animals

All animal procedures were performed in accordance with the Fundamental Guidelines for Proper Conduct of Animal Experiments and Related Activities in Academic Research Institutions under the jurisdiction of the Ministry of Education, Culture, Sports, Science and Technology, Japan. The Animal Care and Use Committee of Hiroshima University approved the experimental protocols. Male ICR mice were obtained from Kyudo (Kumamoto, Japan) and were maintained in a temperature-controlled animal facility with a 12-h light-dark cycle.

### Animal treatment groups

Four-week-old male ICR mice were placed in one of 4 experimental groups and were fed one of the following diets for 28 days: (1) Soybean oil diet. Soybean oil (Nisshin OilliO, Tokyo, Japan) was added to AIN-93G (a standard purified diet without fat, Oriental Yeast Co. Ltd., Tokyo, Japan) at a final concentration of 7% (w/w). (2) Cottonseed oil diet. Cottonseed oil (Nisshin OilliO) was added to AIN-93G at a final concentration of 7% (w/w). (3) Cottonseed oil diet supplemented with DHA. DHA was added to a cottonseed oil diet at a final concentration of 4% (w/w) of total fat. (4) Cottonseed oil diet supplemented with DHA with the administration of letrozole. The mice were fed a cottonseed oil diet supplemented with DHA. Letrozole was suspended in a 0.5% (w/v) methylcellulose 400 and intraperitoneally injected (10 mg/kg) every other day during the intake of the diet. The chow was maintained at −30 °C until use. Experiments shown in Figs 1–3 were performed using (1–3) treatment groups, while those shown in Figs 4 and 5 were done with (1–4) treatment groups.

### Fatty acid content in oils

The NH Foods Ltd. Research and Development Center (Tsukuba, Japan) determined the fatty acid levels in soybean and cottonseed oils using gas chromatography.

### Total RNA extraction and real-time PCR

mRNA levels were determined by methods previously reported^47^. Briefly, total RNA was extracted from microglia using a High Pure RNA Isolation Kit (Roche Diagnostics K.K., Tokyo, Japan). Single-stranded cDNA was synthesized from 1 μg of total RNA according to the ReverTra Ace protocol (Toyobo, Osaka, Japan) with a random primer (9-mer; Takara Bio). A real-time polymerase chain reaction (PCR) was performed using a LightCycler instrument (Roche Diagnostics) with SYBR Green real-time PCR master mix (Toyobo). The primer sequences used in this study are listed in Table 2. mRNA levels were corrected to levels of β-actin mRNA, Relative mRNA levels were calculated by dividing treated mice levels by control mice levels (soybean oil group).

**Table 2:** Primer sequences used in the present study.

	Forward (5′-3′)	Reverse (5′-3′)
StAR	GCTGGAAGTCCCTCCAAGAC	GCCACCCCTTCAGGTCAATA
TSPO	AGAAACCCTCTTGGCATCCG	GCCATACCCCATGGCTGAATA
P450scc	GACACTGAGACTCCACCCCA	CTCGACCCATGGCAAAGCTA
3β-HSD1	CTTGAAGCTGCCCCTGATCT	CTTTATTCCTGTGCAGCAGCC
5α-Red1	ATTTTGGGGAGCTCGTGGAG	GTACCACTGATGATGCTGCCT
P450 17α	GAGTTTGCCATCCCGAAGGA	GAAGCGCTCAGGCATAAACC
17β-HSD3	TATTCAGGTGCTGACCCCTT	ACAAACTCATCGGCGGTCTT
P450arom	GCATGCATGAGAACGGCATC	GGCCCGTCAGAGCTTTCATA
AR	CTCTGGGAGCTCGTAAGCTG	GCTGCCAGCATTGGAGTTTT
ERα	TTCTTCTCAAGCAGGTGGCCC	GCGAGTTACAGACTGGCTCC
ERβ	CCTCGTTCTGGACAGGTCCTC	ATCCCTTGGGACAGCACTCT
PR	CATGGTCCTTGGAGGTCGTA	CTCTCGTTAGGAAGGCCCAC
SOD1	GGAACCATCCACTTCGAGCA	CCCATGCTGGCCTTCAGTTA
SOD2	GTGTCTGTGGGAGTCCAAGG	AGCGGAATAAGGCCTGTTGT
GPx1	CCGGGACTACACCGAGATGA	CCATTCTCCTGGTGTCCGAA
Cat	CTCGCAGAGACCTGATGTCC	GACCCCGCGGTCATGATATT
GR	TGGCACTTGCGTGAATGTTG	CGTGCATGAATTCCGAGTGC
GSTA3	ACAGCTTTTTAACAAGAAAACCCA	TTTCTTCAAACTCCACACCAGC
GSTM1	CCTGCCCACGTTTCTCTAGT	GATCGGGTGTGTCAGTCCG
M-GST	GGTGAAAAGTCCCAGAAGTGC	TCAAATGACTGAATCCAGGGAG
β-actin	CTAGGCACCAGGGTGTGATG	GGGGTACTTCAGGGTCAGGA

### Membrane fractions and immunoblotting

The mouse cerebral cortex was homogenized in buffer (50 mM Tris–HCl, pH 7.4, 154 mM KCl, 1 mM EDTA, 1 mM DTT containing a protease inhibitor cocktail [Nacalai Tesque]) and centrifuged at 10,000 × *g* for 20 min at 4 °C. The supernatant was re-centrifuged at 100,000 × *g* for 60 min at 4 °C. The resulting pellet was lysed with radioimmunoprecipitation assay (RIPA) buffer (25 mM Tris-HCl pH 7.6, 150 mM NaCl, 1% NP-40, 1% sodium deoxycholate and 0.1% SDS) and then used for immunoblotting.

Equal amounts of protein were loaded and separated using sodium dodecyl sulfate polyacrylamide gel electrophoresis (SDS-PAGE) with a 10% or 12% (w/v) polyacrylamide gel and transferred onto a polyvinylidene difluoride membrane. The blocked membranes were incubated with the primary antibodies, anti-P450arom (1/500; Acris Antibodies, Herford, Germany) and anti-α-tubulin (1/2,000; Sigma-Aldrich). The membranes were incubated with peroxide-conjugated secondary antibodies (Thermo Fisher Scientific, Waltham, MA, USA) and then visualized using peroxide substrates (SuperSignal West dura; Thermo Fisher Scientific) in the LAS-1000 Imaging System (Fujifilm, Tokyo, Japan). The results were analyzed with Multi Gauge version 2.1 software (Fujifilm).

### Brain levels of 17β-estradiol

Levels of 17β-estradiol in the cerebral cortex were measured as previously described^12^. Briefly, [^3^H]-17β-estradiol was added to homogenates as an internal standard. The steroid extracts were applied to a C18 Amprep solid-phase column (Amersham Biosciences, Arlington Heights, IL, USA) to remove any contaminating fats. 17β-estradiol was separated using a normal-phase HPLC system (Jasco, Tokyo, Japan) with a silica gel column (Cosmosil 5SL; Nacalai Tesque). The purified 17β-estradiol was quantified using EIA kit (Cayman Chemical, Ann Arbor, MI, USA). To confirm that chemically distinct 17β-estradiol was detected in the EIA, some samples were analyzed by LC-MS/MS at Asuka Pharmamedical Co., Ltd. (Kawasaki, Japan). 17β-estradiol, extracted and purified according to the above procedure, was derivatized with picolinic acid to form estradiol dipicolinoyl ester. The derivatives were purified using a C18 Amprep solid-phase column and then analyzed by LC-MS/MS. The procedure is described previously^48^.

### Seizure induction

Animals were placed in a plastic chamber (15 × 15 × 30 cm). Their behavior was observed before and after the administration of PTZ. Once the animals displayed a resting posture, they were intraperitoneally injected with a convulsive dose of PTZ (60 mg/kg). The control mice received a saline (0.1 ml/10 g) injection. Mice were observed for 15 minutes for convulsive behavior following treatment. Convulsions were classified and scored accordingly^30^: 0, normal; 1, no convulsive behavior; 2, head twitching, myoclonic jerking; 3, clonic convulsion; and 4, kangaroo posture or falling back. Two types of seizures, nonconvulsive (scores 1 and 2) and convulsive motor seizures (scores 3 and 4), occurred after the single-dose administration of PTZ. Acute generalized clonic convulsions, but not tonic convulsions, were induced by a 60 mg/kg dose of PTZ.

### Lipid peroxidation

Thiobarbituric acid-reactive substance (TBARS) was used as an index of lipid peroxidation and measured as described previously^49^. Briefly, the cerebral cortex was homogenized in a 1.15% KCl solution and mixed with SDS, thiobarbituric acid, butylhydroxytoluene and acetic acid buffer. The mixture was boiled for 1 h, and the resulting product was extracted with a 1-butanol-pyridine mixed solution. The absorbance at 532 nm was measured, with 1,1,3,3-tetraethoxypropane serving as a standard.

### Reduced glutathione (GSH) and GSSG

The levels of GSH and GSSG in the cerebral cortex were measured according to methods previously reported^50^. Briefly, the cerebral cortex was isolated and homogenized in 3.5% perchloric acid. After centrifugation, iodoacetic acid was added to the supernatant, and the mixture was neutralized. After incubation for 60 min, 1-fluoro-2,4-dinitrobenzene was added, followed by incubation for 4 h. An aliquot was injected into a μBondapak NH_2_ HPLC column (3.9 × 300 mm; Waters Corporation, Milford, MA, USA) with 80% methanol (solvent A) and 0.5 M acetate buffer in 64% methanol (solvent B) as the mobile phase. The flow rate was 1 mL/min. The gradient of solvent B was as follows: 20% (0–5 min), 20–95% (5–25 min) and 95% (25–45 min). Ultraviolet detection was performed at 350 nm. A known concentration of GSH or GSSG was used as a standard.

### Statistical analyses

All of the data are expressed as the mean ± standard error (S.E.). The statistical analyses were performed using a one-way analysis of variance (ANOVA), followed by Tukey’s test or Student’s t-test. P values of < 0.05 were considered to indicate statistical significance.

## Electronic supplementary material

Supplementary Information
