# Supplementary material for: Potentiation of 17β-estradiol synthesis in the brain and elongation of seizure latency through dietary supplementation with docosahexaenoic acid
Source: Sci Rep. 2017 Jul 24;7:6268. doi: 10.1038/s41598-017-06630-0 (PMC5524681; doi:10.1038/s41598-017-06630-0)

Supplementary Information

Potentiation of 17β-estradiol synthesis in the brain and elongation of seizure latency through dietary supplementation with docosahexaenoic acid

Yasuhiro Ishihara, Kouichi Itoh, Miki Tanaka, Mayumi Tsuji, Toshihiro Kawamoto, Suguru Kawato, Christoph F.A. Vogel and Takeshi Yamazaki

Supplementary Methods

Measurement of letrozole contents in the brain

The cerebral cortex, cerebellum and hippocampus were homogenized in saline to prepare homogenates. Letrozole was extracted with 500 μL of diethyl ether from serum or homogenates. After the inspissation of diethyl ether, the resulting residue was dissolved in 70% methanol and subjected to high-performance liquid chromatography (HPLC) equipped with an ultraviolet light detector. The HPLC conditions were as follows: column, ODS3 250×4.6 mm, 5 μm (GL Sciences, Tokyo, Japan); mobile phase, 70% methanol; flow rate, 1 mL/min; detection, 239 nm. A known concentration of letrozole was used as a standard.

Immunohistochemistry

The brains were removed, post-fixed overnight in 4% buffered PFA at 4 °C after perfusion and cryoprotected in 30% sucrose. Brains were frozen in powdered dry ice, and 50-μm-thick floating sections were prepared using a Cryostat (CM3050 S; Leica Biosystems, Nussloch, Germany). The sections were blocked and permeabilized with PBS including 10% normal goat serum (Sigma-Aldrich) and 0.3% Triton-X 100 for 1 h at room temperature. The sections were incubated with primary antibody (Anti-Iba1, 1/500, Wako; Anti-CD68, 1/200, Serotec/Bio-Rad, Raleigh, NC, USA) for 3 h at room temperature, followed by secondary antibody (Anti-rat IgG, Alexa488, 1/200; Anti-rabbit IgG, Alexa568, 1/200, Molecular Probes-Thermo Fisher

Scientific, Waltham, MA, USA) for 1 h at room temperature in the dark. The sections were mounted on a glass slide with DAPI-Fluoromount-G (Southern Biotech, Birmingham, AL, USA). Images were obtained using a Zeiss LSM700 confocal fluorescence microscope equipped with diode lasers (405, 488, and 568 nm; Carl Zeiss, Oberkochen, Germany). The images were processed using the accompanying Zen image acquisition software package (Carl Zeiss).

Total RNA extraction and real-time PCR

Additional sequences of primers used in the supplemental experiments are shown in Supplementary Table 1.

Legends of Supplementary Figures

Supplementary Fig. S1. Summary of the steroid hormone synthetic pathway in the brain. The abbreviations are defined in the legend of Figure 1.

Supplementary Fig. S2. Effects of fatty diet on (a) body weight, (b) food intake, (c) whole brain weight and (d) cerebral cortex, cerebellum and hippocampus weights. Mice were fed a diet including soybean oil, cottonseed oil or cottonseed oil supplemented with DHA for 28 days. (a) Body weight and (b) food intake were sequentially measured. After 28 days of feeding, the brain was removed, and (c) whole brain weight and (d) cerebral cortex, cerebellum and hippocampus weights were measured. The values are the means ± S.E. (n = 15 - 20 animals in each group).

Supplementary Fig. S3. Changes in letrozole concentrations in the blood and brain. After 10 mg/kg of letrozole was intraperitoneally injected to male ICR mice, blood was collected to isolate the serum, and the brain was removed, followed by dissection. Letrozole levels in the serum, cerebral cortex (CX), cerebellum (CB) and hippocampus (HP) were determined using HPLC and subsequent UV detection. The right upper panel shows an enlarged view. The values are the means ± S.E. (n = 4 animals in each group).

Supplementary Fig. S4. Effects of DHA on microglial morphology and activity. Mice were fed a diet including soybean oil, cottonseed oil or cottonseed oil supplemented with DHA for 28 days. Floating tissue sections, including the cerebral cortex, were prepared, and double-stained with Iba1 and CD68 to evaluate microglial activity. Upper panels show CD68 (green) staining, middle panels show Iba1 staining (red) and bottom panels show CD68 and Iba1 double staining. Results are representative of three independent experiments.

Supplementary Fig. S5. Effects of DHA on the expression of pro-inflammatory molecules and astrocytic markers. Mice were fed a diet including soybean oil, cottonseed oil or cottonseed oil supplemented with DHA for 28 days. The mRNA expression of pro-inflammatory molecules and astrocytic markers in the cerebral cortex was measured by real-time PCR. Levels of mRNA are represented as the fold-change from those in the soybean oil group. The values are the means ± S.E. (n = 9 animals in each group). IL-1β, interleukin-1β; iNOS, inducible nitric oxide synthase; GFAP, glial fibrillary acidic protein.

Supplementary Table S1. Primer sequences for the evaluation of glial activity

	Forward (5′-3′)	Reverse (5′-3′)
IL-1β	AGCTTCCTTGTGCAAGTGTCT	GCAGCCCTTCATCTTTTGGG
iNOS	TCCTGGACATTACGACCCCT	CTCTGAGGGCTGACACAAGG
GFAP	AGGGCGAAGAAAACCGCATC	GGTGAGCCTGTATTGGGACA
S100β	TTCCTGGAGGAAATCAAGGAGC	GGAAGTCACACTCCCCATCC

Supplementary Fig. S1

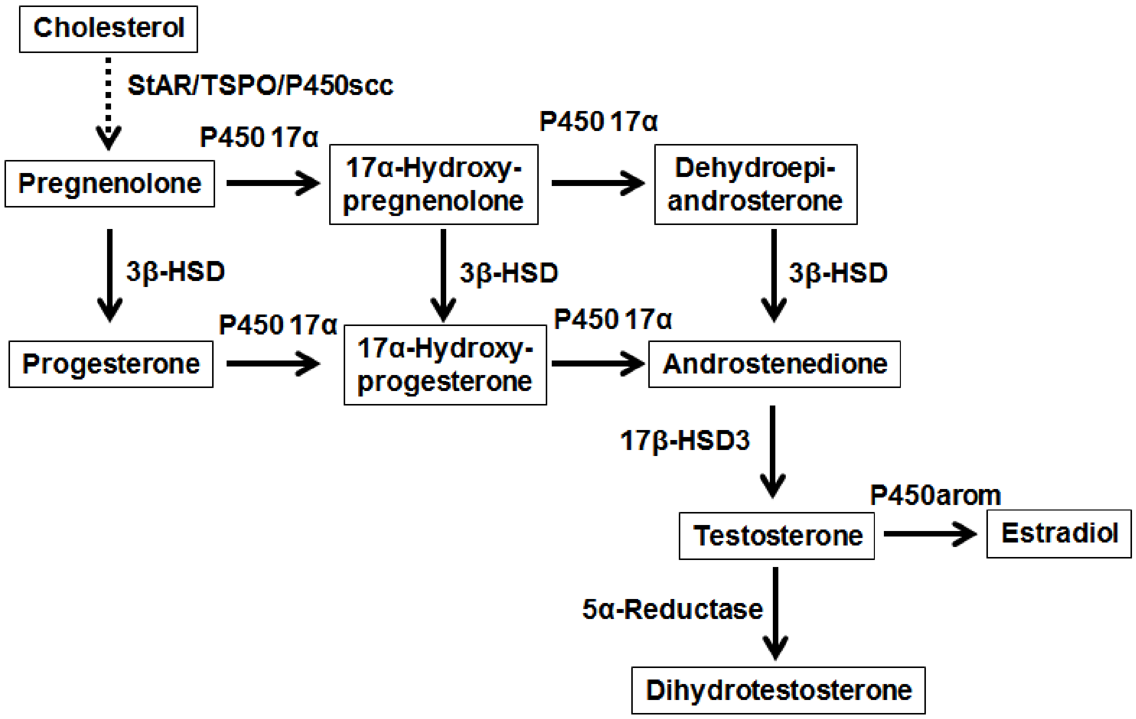

Supplementary Fig. S2

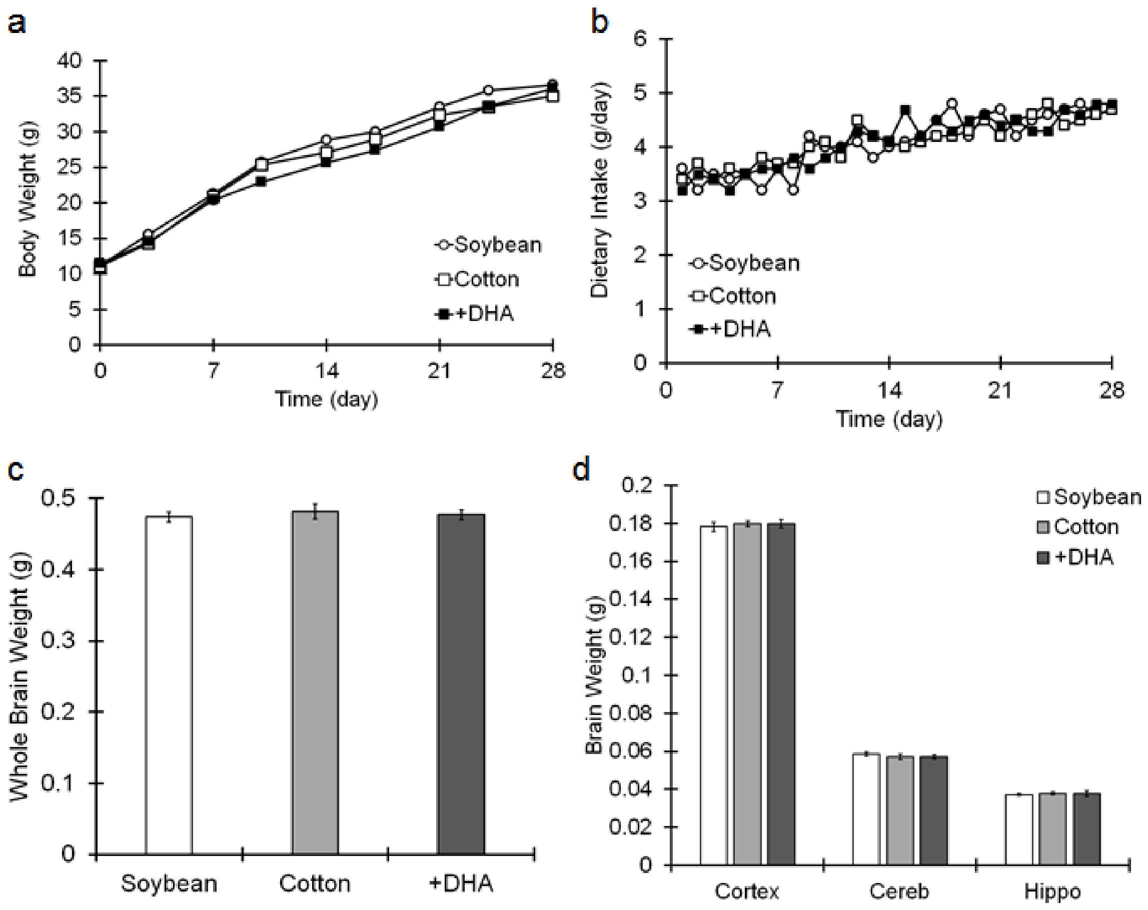

Supplementary Fig. S3

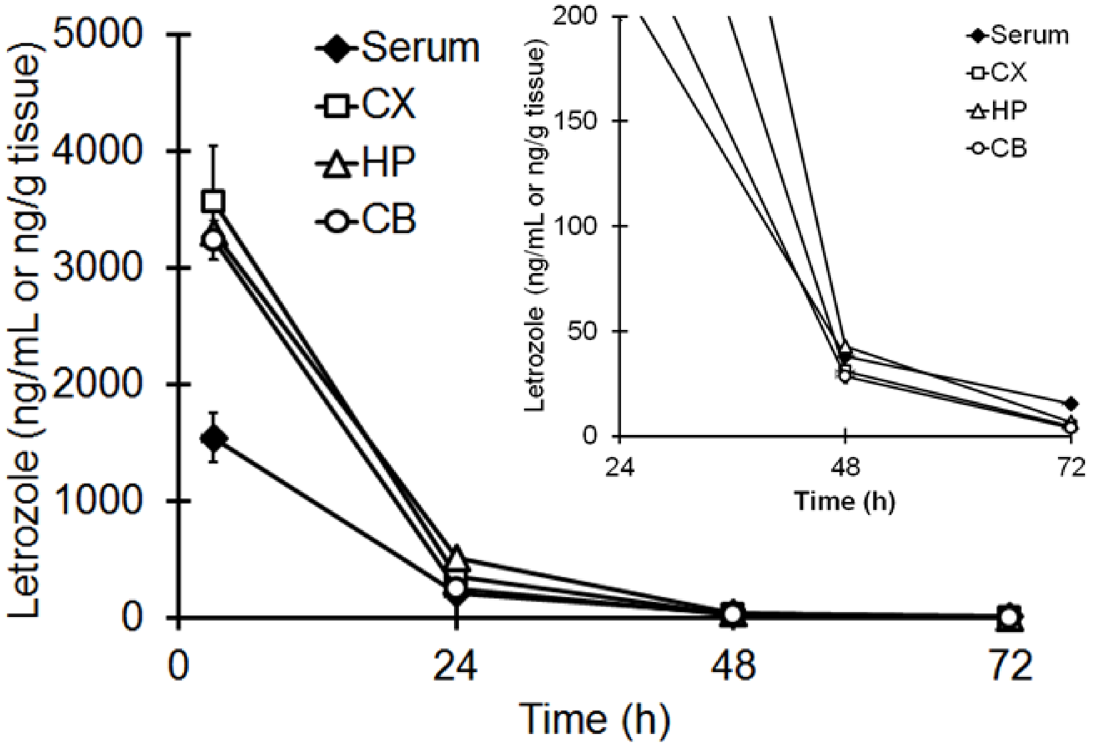

Supplementary Fig. S4

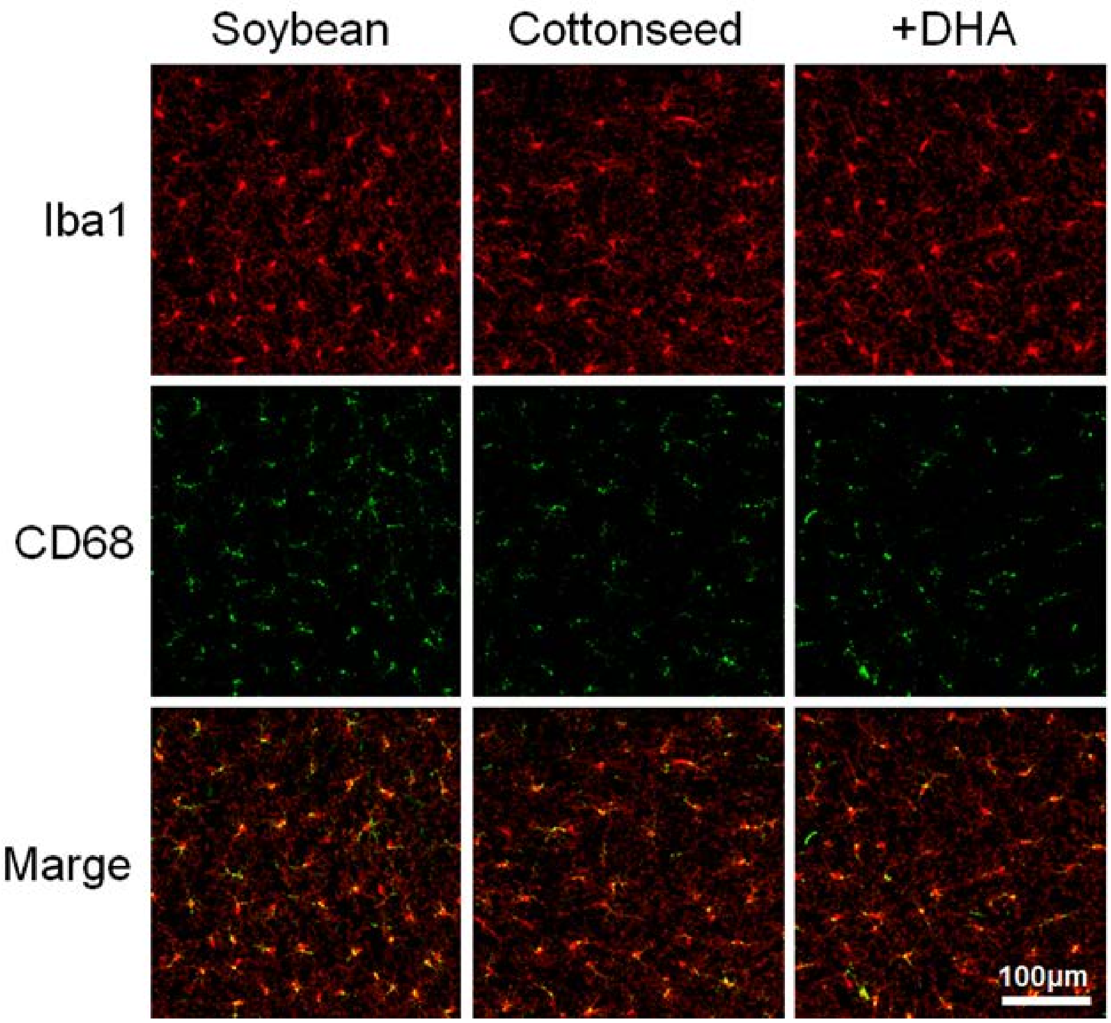

Supplementary Fig. S5

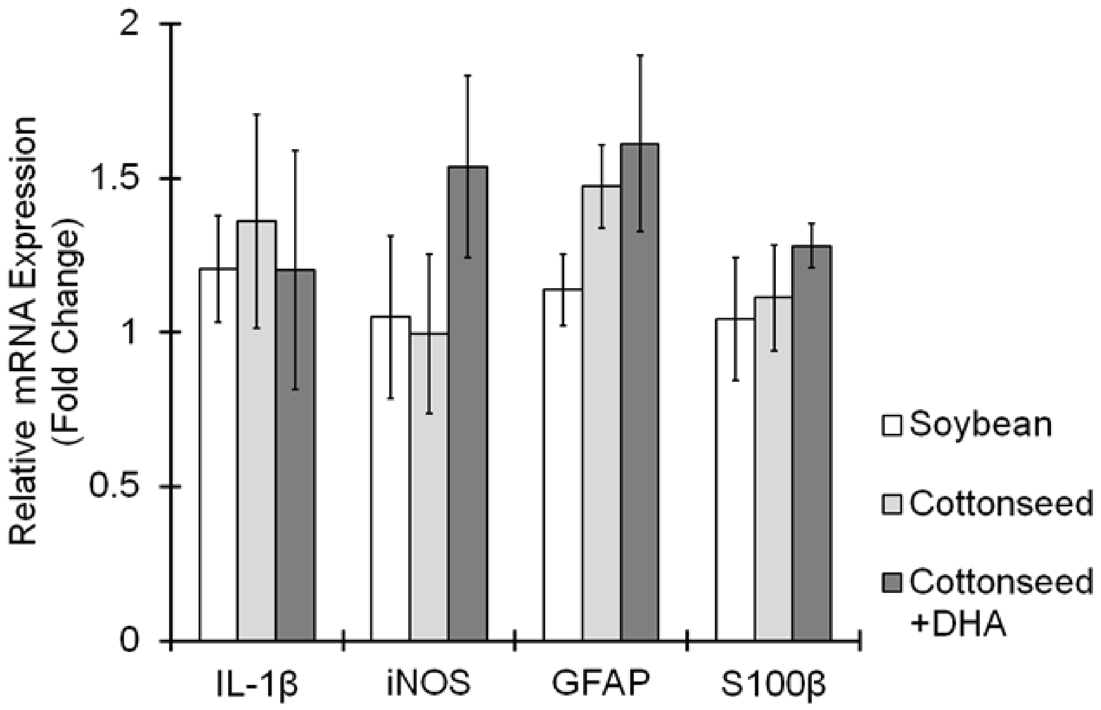

Supplement: Supplementary file 1 — Supplementary Information [file 41598_2017_6630_MOESM1_ESM.pdf]
